# Virus Infection and Death Receptor-Mediated Apoptosis

**DOI:** 10.3390/v9110316

**Published:** 2017-10-27

**Authors:** Xingchen Zhou, Wenbo Jiang, Zhongshun Liu, Shuai Liu, Xiaozhen Liang

**Affiliations:** Key Laboratory of Molecular Virology & Immunology, Institut Pasteur of Shanghai, University of Chinese Academy of Sciences, Chinese Academy of Sciences, Shanghai 200031, China; xczhou@ips.ac.cn (X.Z.); wbjiang@ips.ac.cn (W.J.); zsliu@ips.ac.cn (Z.L.); sliu@ips.ac.cn (S.L.)

**Keywords:** virus infection, death receptor, extrinsic apoptosis, host immune response

## Abstract

Virus infection can trigger extrinsic apoptosis. Cell-surface death receptors of the tumor necrosis factor family mediate this process. They either assist persistent viral infection or elicit the elimination of infected cells by the host. Death receptor-mediated apoptosis plays an important role in viral pathogenesis and the host antiviral response. Many viruses have acquired the capability to subvert death receptor-mediated apoptosis and evade the host immune response, mainly by virally encoded gene products that suppress death receptor-mediated apoptosis. In this review, we summarize the current information on virus infection and death receptor-mediated apoptosis, particularly focusing on the viral proteins that modulate death receptor-mediated apoptosis.

## 1. Introduction of Virus-Mediated Apoptosis

Apoptosis, necroptosis, and pyroptosis are the three major ways of programed cell death (PCD) following virus infection [1,2]. Among them, apoptosis is the most extensively investigated PCD during viral infection. Apoptosis elicited by virus infection has both negative and positive influence on viral replication. Host cells eliminate virally infected cells via apoptosis, which aborts virus infection. On the other hand, some viruses take advantage of inducing apoptosis as a way to release and disseminate progeny viruses [3,4,5]. In both cases, it requires certain viral products to block or delay apoptosis in order to produce sufficient progeny and establish successful viral replication [2,6]. A number of important reviews have provided valuable insights into viruses and apoptosis. This review specifically focuses on viral modulation of death receptor-mediated apoptosis.

## 2. Intrinsic and Extrinsic Apoptosis Pathways

Apoptosis can be triggered by two distinct signaling pathways, namely the intrinsic and extrinsic pathways [7,8,9]. The intrinsic apoptotic pathway is elicited by a wide range of intracellular stress conditions, including cytokine deprivation, DNA damage, oxidative stress, cytosolic Ca^2+^ overload and endoplasmic reticulum stress. These heterogeneous apoptotic signals converge to trigger one pivotal event—mitochondrial outer membrane permeabilization (MOMP), which promotes the release of cytochrome c and other mitochondrial factors into the cytosol, ultimately leading to the generation of initiator and effector caspases and subsequent cell death [7,10]. MOMP is mainly controlled by the B-cell lymphoma 2 (BCL-2) family of proapoptotic proteins. The three BCL-2 homology domains (BH3)-only proteins BCL-2-interacting mediator (BIM) and BH3-interacting domain death agonist (BID) can transiently interact with BCL-2-associated X protein (BAX) or BCL-2 antagonist or killer (BAK) upon activation induced by intrinsic apoptosis signals, leading to the activation and conformational changes of BAX and BAK [11,12]. Activated BAX and BAK allow the formation of high-order homo-oligomers and stable insertion into the outer mitochondrial membranes, promoting MOMP [13,14]. The extrinsic apoptotic pathway is activated by extracellular stress stimulation that is sensed and triggered through activation of death receptors of the tumor necrosis factor (TNF) family, including TNF receptor 1 (TNF-R1), Fas (also called CD95 or Apo-1 or TNFRSF6), TNF-related apoptosis-inducing ligand (TRAIL) receptors (TRAIL-R1 and TRAIL-R2, also known as DR4 and DR5), DR3 and DR6 [7]. Death receptors are type-I transmembrane proteins and are characterized by an extracellular ligand-binding region containing 1-5 cysteine-rich domains, a membrane-spanning region, and a 60- to 80-amino acid cytoplasmic death domain [15,16,17]. Ligand binding to the death receptor on the cell surface leads to signal transduction through the formation of the death-inducing signaling complex (DISC), which mediates the subsequent apoptotic signal transduction [18,19]. Unlike the intrinsic pathway, apoptosis via death receptor-mediated extrinsic pathway does not always require mitochondria. In type I cells without the involvement of intrinsic pathway, the extrinsic apoptotic pathway results in the activation of caspase-8, which can directly induce the activation of caspase-3 and -7, leading to apoptosis [12]. However, both pathways generate similar effector caspases that serve to amplify the initial death signal [20]. In type II cells, the extrinsic pathway can also link to the intrinsic apoptotic pathway via caspase-8 cleavage of BH3-only protein BID [8]. Following death receptor stimulation, activated caspase-8 cleaves BID into 15 kDa truncated form tBID. tBID then triggers MOMP and cytochrome C release, initiating effector caspase activation and apoptosis [21].

Signaling through both Fas and TRAIL-R1/-R2 leads to the oligomerization of death receptors and intracellular assembly of DISC composing of dead receptor, the adaptor molecule FADD (FAS-associated with a death domain), procaspase-8, procaspase-10 and cellular FADD-like interleukin-1β converting enzyme (FLICE)-like inhibitory protein (c-FLIP). c-FLIP has two protein isoforms, c-FLIP long (c-FLIP_L_) and c-FLIP short (c-FLIP_S_) [22]. c-FLIP isoforms control the activation of caspase cascade that emanates from caspase-8 and then initiate the apoptotic program or nonapoptotic caspase-8 signaling [23,24,25]. In contrast to Fas and TRAIL death receptors, TNF-R1-mediated apoptotic signaling is more complex. TNF-α binding to TNF-R1 recruits TNFR-associated protein with death domain (TRADD) as an adaptor protein which subsequently recruits FADD, TNF-associated factor-2 (TRAF-2), receptor-interacting protein (RIP), and RIP-associated interleukin-1 beta-converting enzyme homolog (ICH-1)/cell death protein-3 (CED-3)-homologous protein with a death domain (RAIDD). FADD then binds and activates caspase-8 and -10, leading to apoptosis [17,26].

## 3. Viral Induction and Viral Activators of Death Receptor-Mediated Apoptosis

**Figure 1:**
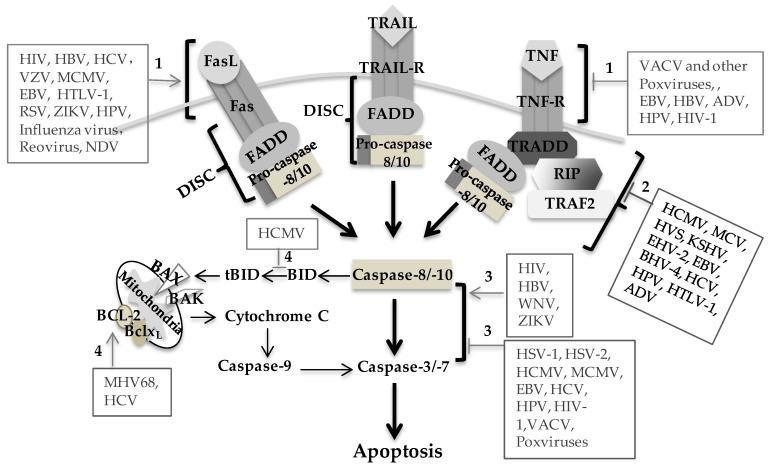
Viral modulation of death-receptor mediated apoptosis. Death receptors Fas, TRAIL-R and TNF-R form DISC upon binding to their ligands, activate caspase cascade and subsequently initiate extrinsic apoptosis. Caspase-8 activation can cleavage BID to tBID and link to mitochondria-mediated intrinsic apoptosis pathway. Virus infection regulates death receptor-mediated extrinsic apoptosis mainly through virally encoded proteins. The regulatory mechanisms involve: (1) regulating the expression and function of death receptors/ligands; (2) interfering DISC formation and function; (3) regulating caspase activities; (4) regulating the expression and function of pro-apoptotic and anti-apoptotic proteins. Black arrow represents signal induction; grey arrow represents signal induced by viruses; grey T bar represents signal inhibited by viruses.

Death receptor-mediated apoptosis represents an efficient mechanism by which the virus can induce cell death and disseminate progeny, which plays an important role in viral pathogenesis and provides a potential therapeutic target. Regulation of death receptor-mediated apoptosis by the virus is mainly through upregulation of death receptors or their ligand on the cell surface of the infected individuals and increased sensitivity of the cells to death receptor-mediated apoptosis (Figure 1). Many viruses encode viral proteins to regulate death receptor-mediated apoptosis in a variety of different ways (Table 1).

### 3.1. Human Immunodeficiency Virus (HIV)

Apoptosis mediated by death receptors plays an important role during HIV-1 infection. An increased expression of Fas antigen in CD4+ and CD8+ T lymphocytes occurs in patients infected with HIV-1, rendering those cells sensitive to FasL/Fas system-mediated apoptosis and contributing to T lymphocyte depletion in HIV-infected individuals [99,100,101,102,103,104,105]. HIV-1 infection also upregulates FasL expression in macrophage and mediates apoptosis and depletion of T lymphocytes [106].

Regulation of TRAIL expression and TRAIL-mediated apoptosis during HIV-1 infection has been well demonstrated. HIV-1 infection induces expression of TRAIL and DR5 and leads to TRAIL-mediated apoptosis in primary CD4+ T cells, which is regulated by IFN-α that is produced by HIV-1-stimulated plasmacytoid dendritic cells (pDCs) [107,108]. Upregulation of TRAIL in primary macrophages during HIV-1 infection occurs and mediates apoptosis in bystander T cells and neuronal cells [27,109,110]. An elevated level of plasma TRAIL was observed in HIV-infected patients and preferentially provokes apoptosis of HIV-1-infected monocyte-derived macrophages and partially mediates CD4+ T-cell apoptosis [111,112]. Additionally, HIV infection results in TRAIL expression and TRAIL-mediated apoptosis in memory B cells, leading to the loss of memory B cells [113]. As such, Fas- or TRAIL-mediated signaling could be exploited for the development of therapeutic target aimed at the prevention of T cell death in AIDS and preventive HIV vaccine.

HIV-1-encoded proteins modulate death receptor-mediated apoptosis in different cell types. HIV-1 Tat, Vpu, gp120 and gp160 proteins sensitize T cells to Fas-mediated apoptosis with different regulatory mechanisms, possibly contributing to T-cell depletion in AIDS [28,31,34]. HIV-1 gp120 accelerates the apoptosis of human lamina propria T cells induced by Fas-mediated activation which is related to increased induction of FasL mRNA [32], whereas gp160 enhancement of Fas-mediated apoptosis involves the activation of caspase-3 and requires calmodulin binding to the C-terminal binding domain of gp160 [34]. The Env protein of CCR5 tropic HIV strains activates Fas and caspase-8 as well as triggers FasL production, which eventually results in CD4+ T cell apoptosis [30]. Furthermore, HIV-1 Tat upregulates TRAIL in peripheral blood mononuclear cells (PBMCs) and primary macrophages, leading to apoptosis and depletion of uninfected bystander cells [27,29]. A recent report indicates that soluble gp120 shed from HIV-1-infected cells and virus infection itself induces TNF-α expression on macrophages, and upregulates TNF-receptor 2 (TNF-R2) expression on the surface of CD8+ T cells. However, whether T-cell death occurs when these two cell populations interact is unexpected, because reports of apoptosis by TNF-R2 signaling are rare [33].

### 3.2. Hepatitis Viruses

In chronic hepatitis C virus (HCV) infection, enhanced hepatocyte apoptosis and upregulation of the death receptors and death-inducing ligands have been described [114,115,116]. Fas expression on PBMCs of HCV-infected patients increases significantly compared with the cells from normal subjects [117]. HCV infection sensitizes human hepatocytes to TRAIL-induced apoptosis in a caspase 9-dependent manner through upregulating DR4 and DR5 [118,119]. Fas- and TRAIL-mediated apoptosis of hepatocytes triggered by viral infection appears to correlate with liver pathology and contributes to fibrogenesis [114,120]. Hepatitis B virus (HBV) replication can also enhance TRAIL-mediated apoptosis in human hepatocytes, in part, by HBV-encoded antigen (HBxAg)-dependent upregulation of TRAIL-R1/DR4 [121].

The pro- and anti-apoptotic roles of HCV proteins are controversial and dependent on the experimental system used [122]. HCV core protein increases the sensitivity of Jurkat T cells to Fas-mediated apoptosis by binding to the cytoplasmic domain of Fas and potentially enhancing the downstream signaling event of Fas-mediated apoptosis [37]. The core protein induces apoptosis in a target T cell expressing Fas, which is mediated by FasL that is upregulated in hepatoblastoma cell line [38]. It also enhances TNF-induced apoptosis by binding to the cytoplasmic domain of TNF-R1 [39]. Additionally, it increases TRAIL-mediated apoptotic cell death in hepatocellular carcinoma cell line, which is dependent on the activation of mitochondria apoptosis signaling pathway [40]. The impact of HBV viral products on death receptor-mediated apoptosis is less clear. HBV X protein (HBX) has been shown to increase DR5 expression through NF-κB pathway and sensitize TRAIL-induced apoptosis in hepatocytes by inhibiting the E3 ubiquitin ligase A20. A20 negatively regulates caspase-8 cleavage and activation through mediating RIP1 polyubiquitination [35,36].

### 3.3. Herpesviruses

Fas antigen expression significantly increases on PBMCs obtained from varicella-zoster virus (VZV) seropositive donors after culture with VZV antigen. The cultured cells undergo Fas-mediated apoptosis, suggesting a potential role of Fas-mediated apoptosis in the elimination of lymphocytes activated by VZV infection [123]. Another report shows that VZV-induced apoptosis activates caspase-8 in human melanoma cells [124]. Murine cytomegalovirus (MCMV) infection increases Fas expression and Fas-mediated apoptosis, leading to reduced number of hematopoietic progenitor cells and contributing to CMV-induced myelosuppression [125], whereas latent infection of myeloid progenitors by human CMV (HCMV) are refractory to Fas-mediated killing through the cellular IL-10/PEA-15 pathway, and HCMV infection in fibroblasts suppresses Fas expression and protects the cells against Fas-mediated apoptosis through de novo virus-encoded gene expression [126,127]. Epstein–Barr virus (EBV)-infected cells release Fas ligand in exosomal fractions and induce FasL-mediated extrinsic pathway in a number of different cell types including B cells, T cells and epithelial cells [128]. EBV also induces Fas expression in CD4+ T cells and FasL expression in B cells and macrophage, which leads to EBV-stimulated T cells undergoing apoptosis [129]. Both EBV latent membrane protein 1 (LMP1) and protein 2A (LMP2A) sensitize the infected B cells to Fas-mediated apoptosis through the increase of Fas expression, susceptible to elimination by the immune system [41,42].

### 3.4. Other RNA Viruses

Influenza virus infection activates Fas gene expression and induces apoptosis of infected cells [130,131,132]. Furthermore, influenza virus infection induces co-expression of Fas and FasL on the surface of infected cells, which causes apoptosis when the infected cells come into contact with each other [133]. Additionally, influenza virus infection increases TRAIL and receptor DR5 expression which plays an important role in the virus clearance by the immune response [134]. Respiratory syncytial virus (RSV) infection in the epithelial cells and other primary airway cells induces extrinsic cell death through an increase of Fas expression and upregulation of TRAIL and its receptors DR4 and DR5 [135,136]. Similarly, reovirus-induced apoptosis is also mediated by the increase of TRAIL release and expression of DR4 and DR5 [137]. Reovirus infection sensitizes different types of cancer cell lines to TRAIL-mediated apoptosis in a caspase 8-dependent manner or through inhibition of NF-κB activation [138,139]. Newcastle disease virus (NDV) infection triggers upregulation of TNF-α and TRAIL which initiate extrinsic apoptosis [140]. Chandipura virus induces neuronal death through the Fas-mediated extrinsic apoptotic pathway [141]. One report shows that dengue virus-induced apoptosis involves in FasL/Fas pathway in vascular endothelial cells [142]. West Nile virus (WNV) infection activates death-receptor-mediated apoptosis in the brains of infected animals through upregulation of caspase activity, which in turn contributes to WNV-induced neuronal injury and pathogenesis [143]. Zika virus (ZIKV) infection of neuronal cells can increase TNF-α expression and activate caspase-3/-7, -8 and -9, which might contribute to ZIKV-induced neuronal cell death and neurotoxicity [144,145]. Both Fas- and TNF-α-mediated cell death signaling play a role in Ebola virus (EBOV)-induced lymphocyte apoptosis, which might contribute to lymphopenia in the infected patients [146,147,148]. Neurovirulent strain of Sindbis virus infection induces TNF-α-mediated apoptosis in PC-12 cells [149].

### 3.5. Other DNA Viruses and Retroviruses

Human papillomavirus (HPV) E2 protein induces apoptosis mediated by FasL and TNF-α in HPV-positive and negative cervical cancer cell lines through interacting with c-FLIP and abrogating the apoptosis-inhibitory function of c-FLIP [43]. HPV E7 expression in genital keratinocytes can also sensitize the cells to TNF-mediated apoptosis [44]. Human T-cell leukemia virus-I (HTLV-1) Tax oncoprotein stimulates NF-κB-dependent expression of TRAIL mRNA and induces TRAIL-mediated T cell death [45]. Likewise, lyssavirus, which is a member of the Rhabdoviridae family, induces TRAIL-dependent apoptosis in neuroblastoma cells through the release of a soluble, active form of TRAIL by encoded matrix protein [46].

## 4. Viral Inhibitors of Death Receptor-Mediated Apoptosis

Elimination of infected cells via death receptor-mediated apoptosis is one of the defense mechanisms against virus infection. Induction of early cell death would severely limit virus production and reduce or eliminate the spread of progeny virus in the host. Thus, many viruses have evolved many different strategies to interfere with death receptor signaling and prevent apoptosis through virally encoded antiapoptotic factors (Table 1), thereby allowing for the production and spread of progeny virus. Some viruses express death receptor orthologs and specifically target death receptors to inhibit apoptosis. The secreted TNF-R2 ortholog of Shope fibroma virus (rabbit poxvirus) can neutralize TNF as a soluble decoy receptor, which is one of the first-described evasion strategies [47,48]. The poxvirus-encoded TNFR ortholog T2 protein and vaccinia virus (VACV)-encoded TNFR ortholog CrmE inhibit TNF-mediated apoptosis of infected cells [49,55]. HCMV also contains a TNFR ortholog encoded by the UL144 gene, but its functional significance remains obscure [150]. Most viral proteins block death receptor-mediated apoptosis mainly through regulation of death receptors or their ligand expression, interaction with apoptotic signaling molecules and interfering with signaling pathways (Figure 1).

### 4.1. Herpesviruses

Herpesviruses have been most instructive for viral inhibitors of death receptor-mediated apoptosis [151]. Herpes simplex virus-1 (HSV-1) glycoprotein D (gD) exhibits NF-κB-dependent protection against Fas-mediated apoptosis in U937 monocytoid cells, which is associated with decreased levels of caspase-8 activity and upregulation of antiapoptotic proteins [57]. The ribonucleotide reductase R1 subunits of HSV-1 and HSV-2 protect cells against FasL-induced apoptosis by interacting with caspase-8 [58]. The HCMV protein IE2 induces the expression of c-FLIP in human retinal pigment epithelial cells and contributes to protection from Fas- and TRAIL-mediated apoptosis [59], whereas HCMV-encoded viral mitochondria-localized inhibitor of apoptosis (vMIA), a product of the viral *UL37* gene, inhibits Fas-mediated apoptosis at a point downstream of caspase-8 activation and Bid cleavage [60]. HCMV UL36 and MCMV homologous protein M36 inhibit Fas-mediated apoptosis through prevention of caspase-8 activation by binding to pro-caspase-8 [61,62]. MCMV-encoded M45 blocks TNF-induced apoptosis through the binding of M45 to the TNFR adaptor protein RIP1 in a manner that is independent of caspase activation [63]. Additionally, M45 also inhibits TNF-α-dependent necrosis by targeting RIP3 and disrupting RIP1–RIP3 interaction [152].

Like poxvirus molluscum contagiosum virus (MCV)-encoded MC159 protein which is a viral FLICE-inhibitory protein (v-FLIP) with two death effector domains and inhibits both Fas- and TNFR-mediated apoptosis [66], several gamma-herpesviruses including herpesvirus saimiri (HVS), Kaposi sarcoma-associated virus (KSHV), equine herpesvirus 2 (EHV-2) and bovine herpesvirus 4 (BHV-4) also encode the v-FLIP. These v-FLIP proteins protect against apoptosis induced by Fas, TNF-R1, and TRAIL-R through interaction with FADD and prevention of procaspase-8 maturation [64,65,66,67]. EBV-encoded small nonpolyadenylated RNA (EBER) protein confers resistance to Fas-mediated apoptosis by blocking protein kinase PKR activity in intestine 407 cells [68,69]. EBV-encoded BHRF1 protein with distant homology to BCL-2 inhibits TNF- and Fas-mediated apoptosis in a cell type-specific manner; the protective mechanism of BHRF1 against apoptosis resembles that of BCL-2 and Bcl-XL as it inhibits activation of cytosolic phospholipase A2 and caspase-3 [70]. However, BHRF1 inhibits TRAIL-induced apoptosis in BJAB cells by functioning downstream of Bid cleavage and upstream of mitochondrial damage [69]. EBV BZLF1 prevents TNF-α activation of target genes and TNF-α-induced apoptosis by downregulating TNFR1 [71]. EBV LMP1 expression confers partial resistance to Fas-mediated apoptosis by reducing caspase activity in BJAB cells [72], and it inhibits TRAIL-mediated apoptosis through activation of PI3K/Akt and expression of c-FLIP in nasopharyngeal carcinoma cells [73]. The murine gammaherpesvirus-68 (MHV68) M11 encodes a BCL-2 ortholog which inhibits Fas- and TNF-α-mediated apoptosis [74].

### 4.2. Hepatitis Viruses

HCV also encodes several proteins that antagonize host cell death signals. Although HCV core protein sensitizes Jurkat T cells to Fas-mediated apoptosis, it inhibits Fas-mediated apoptosis via NF-κB activation in particular HepG2 cell lines, suggesting its cell type-specific function [75]. The core protein blocks TNF-α-mediated apoptosis through inhibition of caspase-8 activation by sustaining c-FLIP expression and proteolytic cleavage of the death substrate poly (SDP-ribose) polymerase [76,77]. HCV E2 protein activates phosphorylation of IkBα, increases the expression of antiapoptotic BCL-2 family proteins, and confers Raji cells and primary human B lymphocytes protection against Fas-mediated apoptosis [78]. HCV non-structural protein 5A (NS5A) impairs TNF-mediated apoptosis by interfering the association between TRADD and FADD [79]. HBV core protein prevents Fas-mediated apoptosis by regulation of Fas and FasL expression [80].

### 4.3. Adenoviruses

The E3 region of adenoviruses (ADV) encodes several proteins that modulate death receptors on the cell surface and death receptor-mediated apoptosis. The E3-10.4K/14.5K complex selectively mediates loss of Fas surface expression and blocks Fas-induced apoptosis of virus-infected cells [81], whereas the E3 proteins, 6.7K, 10.4K and 14.5K complex, can induce downregulation of TRAIL-R1 and TRAIL-R2 from the cell surface and block the infected cells from TRAIL-mediated apoptosis [82]. The E3 receptor internalization and degradation (RID) complex prevents apoptotic cell death initiated through dead receptors including TNF-R1, TRAIL-R1, and Fas [83]. Adenovirus type 5 encoded 14.7 kDa inhibits Fas-mediated apoptosis through interaction with FLICE and TNF-mediated apoptosis by inhibiting TNF-R1 internalization and DISC formation [84].

### 4.4. Human Papillomaviruses

High-risk HPV type 16 (HPV16) and 18 (HPV18) play a pivotal role in the pathophysiology of cervical cancer. Like other viruses, HPV has also developed strategies to block host-mediated apoptosis and regulate the survival of infected cells [153]. Some evidence suggests that the oncoproteins of HPV and E5 can inhibit death receptor signaling pathway by different mechanisms [85,86]. E5 inhibits Fas-induced apoptosis, in part, by decreasing the cell surface expression of the Fas receptor whereas E5 inhibits TRAIL signaling by interfering with the formation of TRAIL DISC and subsequent cleavage of procaspases-8 and -3, as well as of PARP [85]. The E6 oncoprotein of HPV can inhibit TNF-mediated apoptosis through interacting with the death domain of the TNF-R1 and blocking TNF-R1 interaction with TRADD in mouse fibroblasts, human monocytes/histocytes, and osteosarcoma cells [88,89,90]. The E6 protein can also protect TRAIL-induced apoptosis by facilitating the degradation of FADD and caspase-8 [87]. The E7 oncoprotein of HPV inhibits TNF-mediated apoptosis in keratinocytes by upregulation of antiapoptotic protein c-IAP2 [92]. The mechanism of E7 in delaying Fas-mediated apoptosis and preventing TNF-mediated apoptosis is also involved in the suppression of caspase-8 activation [91].

### 4.5. Other Viruses

Glycoproteins of EBOV and Marburg virus (MARV) suppress Fas-mediated apoptosis in Hela cells [93]. HIV-1 Nef expression confers resistance against Fas-mediated apoptosis through inhibition of caspase-3 and caspase-8 activation [94], whereas HIV-1 Tat protects Jurkat T cells from TRAIL-mediated apoptosis [95]. HTLV-1 transactivator protein Tax inhibits Fas-mediated apoptosis by induction of c-FLIP through activation of NF-κB [96,97]. HTLV-2 Tax protein also inhibits Fas-mediated apoptosis, but the mechanism remains unclear [98]. Poxviruses encode conserved serine protease inhibitors (serpins) which inhibit caspase-8 activity and Fas- and TNF-mediated apoptosis, such as CrmA protein of cowpox virus, SPI-2 of rabbitpox, vaccinia, variola and ectromelia viruses, and SPI-1 protein of vaccinia virus [50,51,52,53,54,56].

## 5. Consequence of Death Receptor-Mediated Apoptosis during Viral Infection

For many viruses, induction of apoptosis during lytic infection or at late stages of infection may be an important step for the dissemination of progeny virus to neighboring cells while also evading host immune inflammatory and immune responses. With some viruses, inhibition of apoptosis in virus-infected cells can prevent premature death of the host cell and impair virus production, which enables the establishment of viral latency and facilitates persistent infection, contributing to the avoidance of immune surveillance by the host. Therefore, in certain circumstance, either induction or inhibition of death receptor-mediated apoptosis could assist viral infection and contribute to viral pathogenesis.

For the host, death receptors can be mediators of the innate immune response to viral infection. The murine and human TRAIL promoters contain interferon regulatory elements and can be activated by interferons, and thus TRAIL is one of the earliest genes induced by interferons [154,155]. Many innate immune cells increase TRAIL expression by proinflammation cytokines like interferons that are produced during viral infection. TRAIL-mediated apoptosis thus could play a role in the clearance of virus-infected cells by innate immune cells, especially natural killer (NK) cells. NK cells express the TNF family of cytokines and mediate cytotoxicity through the TRAIL/TRAIL-R signaling and granzyme/perforin mechanisms [155]. TRAIL expression on NK cells can be induced by other cytokines and has been shown to involve in the killing of activated NK cells against virus-infected cells [156]. For instance, IFN-α- or IL26-induced TRAIL expression on NK cells is associated with antiviral cytotoxicity of NK cells and the control of HCV infection in chronic HCV-infected patients [157,158]. Similarly, IFN-α/β-induced modulation of the TRAIL/TRAIL-R system enhances the NK cell-mediated apoptotic killing of murine cells infected with encephalomyocarditis virus [155]. Besides, NK cells can eliminate virus-specific T cells through TRAIL-mediated apoptosis. Such as, NK cells rapidly eliminate HBV-specific T cells which display high-level expression of TRAIL-R2 in patients with chronic hepatitis B and activated CD4+ T cells in the salivary gland during chronic MCMV infection [159,160]. However, some viral proteins can antagonize NK-mediated killing through modulation of TRAIL/TRAIL-R system. HCMV glycoprotein UL141 binds to TRAIL-R2 and thus protects virus-infected cells from TRAIL and TRAIL-dependent NK cell-mediated killing [161,162]. MCMV m166 open reading frame inhibits expression of TRAIL-DR in infected cells and thus thwarts NK-mediated killing [163]. Apart from its important role in NK cell killing activity, TRAIL-mediated apoptosis is also involved in the cytotoxicity of pDCs. Measles virus and influenza virus can induce TRAIL expression on the surface of pDC and enable the cytotoxic killing of pDC against TRAIL-sensitive target cells [164,165]. One study reports that HIV-1 viremia is associated with the upregulation of TRAIL-R1 on activated CD4+ T cells which become susceptible to TRAIL-dependent pDC-mediated killing [166].

In addition to the role in the cytotoxic activity of innate immune NK cells and pDC cells, death receptor-mediated apoptosis plays an important role in the cytotoxic T cell killing during viral infection. It is well demonstrated that some virus-specific cytotoxic T lymphocytes (CTLs) use the FasL/Fas-dependent lytic mechanism to kill virus-infected or bystander cells, such as lymphocytic choriomeningitis virus (LCMV)-infected cell lysis by LCMV-specific CD4+ CTL [167], MHC class I-restricted killing of neurons by LCMV-specific CD8+ T lymphocytes [168], Ag-bearing cell killing and non-Ag-bearing bystander cell killing by HCV-specific CTLs [169,170], and growth inhibition of EBV- or MHV68-infected B cells by virus-specific CTLs [171,172,173]. In addition, Fas- and TRAIL-mediated apoptosis regulate clearance of influenza A virus (IAV) by IAV-specific CD8+ T cells [174,175]. Conversely, Fas-mediated apoptosis can also cause the elimination of some virus-specific CTLs, such as HIV-, HCV- and EBV-specific CTLs [176,177,178]. The sensitivity of CTLs to Fas-induced apoptosis is of particular importance for the virus as it impairs the capability of virus-specific CTLs to kill virus-infected cells, thus resulting in the escape of virally infected cells from the CTL response.

Death receptors also mediate apoptosis-independent processes during viral infection. For instance, FasL/Fas system participates in the induction of inflammatory response during virus infection. This has been mainly demonstrated in the context of HSV-2 infection, during which it regulates inflammation in vaginal tissue via the Fas/FasL pathway [179,180,181]. This content is not within the focus of this review and would not be further discussed here.

## 6. Concluding Remarks

Death receptor-mediated apoptosis represents a complex and co-evolved mechanism used by the virus and the host, which contributes to viral pathogenesis and host immune surveillance. The infected host cell uses it as part of the antiviral response, whereas the virus appears to balance apoptotic and anti-apoptotic effect to facilitate viral infection. With respect to the potential use of death receptor-mediated apoptosis in the treatment of viral diseases, therapeutic strategies to enhance death receptor-mediated apoptotic clearance of virus-infected cells may be beneficial in some viral infections, whereas in viral infections in which pathogenesis and propagation are enhanced by apoptosis, inhibition of death receptor-mediated apoptosis may be the therapeutic goal. Furthermore, death receptor-mediated apoptosis plays a critical role in the control of virus-infected cells by NK cells, pDCs, and CTLs, which could be the basis for the development of targeted immune control of virus infection. Future studies will need to elucidate in more detail the mechanisms of death receptor-mediated apoptosis by which those immune cells mediate antiviral function. Viral products involved in the induction and suppression of death receptor-mediated apoptosis provide critical insights into cellular apoptotic processes, which could be useful in treating viral diseases. Understanding the mechanism of virally induced death receptor-mediated apoptosis is vital because of its involvement in the pathophysiology of diseases and therapeutic intervention. Given the multifaceted role of death receptor-mediated apoptosis, further preclinical and clinical studies are required in order to determine its specific usage in the treatment of viral diseases.

## Figures and Tables

**Table 1:** Viral proteins that modulate death receptor-mediated apoptosis.

**Virus**	**Viral Activator**	**Mediated Signaling**	**References**
HIV-1	Tat	Fas, TRAIL-R	[27,28,29]
	Env	Fas	[30]
	Vpu	Fas	[31]
	gp120	Fas, TNF-R	[28,32,33]
	gp160	Fas	[34]
HBV	HBX	TRAIL-R	[35,36]
HCV	Core protein	Fas, TNF-R, TRAIL-R	[37,38,39,40]
EBV	LMP2A	Fas	[41]
	LMP1	Fas	[42]
HPV	E2	Fas, TNF-R	[43]
	E7	TNF-R	[44]
HTLV-1	Tax	TRAIL-R	[45]
Lyssavirus	Matrix protein	TRAIL-R	[46]
**Virus**	**Viral Inhibitor**	**Mediated Signaling**	**References**
Fibroma virus	TNFR2 ortholog	TNF-R	[47,48]
Vaccinia virus	CrmE	TNF-R	[49]
	SPI-1	Fas	[50]
	SPI-2	Fas, TNF-R	[50,51,52,53,54]
Myxoma virus	T2	TNF-R	[55]
Cowpox virus	CrmA	Fas, TNF-R	[56]
HSV-1	gD	Fas–	[57]
	Ribonucleotide reductase R1	Fas	[58]
HSV-2	Ribonucleotide reductase R1	Fas	[58]
HCMV	IE2	Fas, TRAIL-R	[59]
	vMIA	Fas	[60]
	UL36	Fas	[61]
MCMV	M36	Fas	[62]
	M45	TNF-R	[63]
KSHV	v-FLIP	Fas, TRAIL-R	[64,65]
MCV	MC159	Fas, TNF-R, TRAIL-R	[65,66,67]
EHV2	E8	Fas, TNF-R	[66]
HVS	v-FLIP	Fas, TRAIL-R	[65]
BHV-4	v-FLIP	Fas, TRAIL-R	[65]
EBV	EBER	Fas	[68,69]
	BHRF1	Fas, TNF-R, TRAIL-R	[70,69]
	BZLF1	TNF-R	[71]
	LMP1	Fas, TRAIL-R	[72,73]
MHV68	M11	Fas, TNF-R	[74]
HCV	Core protein	Fas, TNF-R	[75,76,77]
	E2	Fas	[78]
	NS5A	TNF-R	[79]
HBV	Core protein	Fas	[80]
ADV	E3-10.4K/14.5K complex	Fas	[81]
	E3-6.7K/10.4K/14.5K complex	TRAIL-R	[82]
	E3-RID complex	Fas, TNF-R, TRAIL-R	[83]
	E3-14.7K	Fas, TNF-R	[84]
HPV	E5	Fas, TRAIL-R	[85,86]
	E6	TNF-R, TRAIL-R	[87,88,89,90]
	E7	Fas, TNF-R	[91,92]
EBOV	Glycoprotein	Fas	[93]
MARV	Glycoprotein	Fas	[93]
HIV-1	Nef	Fas	[94]
	Tat	TRAIL-R	[95]
HTLV-1	Tax	Fas	[96,97]
HTLV-2	Tax	Fas	[98]
